# Anti‐Doping Testing at FIFA World Cup 2022

**DOI:** 10.1002/dta.3868

**Published:** 2025-02-18

**Authors:** Alanod D. AlQahtani, Wadha Abushareeda, Ariadni Vonaparti, Suhail Kraiem, Wesal ElSaftawy, Khadija Saad, Aisha Al‐Wahaibi, Nayla Hilal, Najib Dbes, Vandana Nimker, Alexis Weber, Alka Beotra, Mohammed Al Maadheed

**Affiliations:** ^1^ Anti‐Doping Lab‐Qatar (ADLQ) Doha Qatar; ^2^ Institute of Doping Analysis and Sports Biochemistry (IDAS) – Dresden Kreischa Germany; ^3^ Medicine & Science, Federation Internationale de Football Association Zürich Zurich Switzerland; ^4^ Centre for Metabolism and Inflammation, Division of Medicine University College London London UK

**Keywords:** anti‐doping lab Qatar, doping analysis, FIFA, Qatar, WADA, World Cup 2022

## Abstract

The 22nd FIFA World Cup, the world championship for national football teams, was held in Qatar from November 20th to December 18th, 2022. The doping control analysis was successfully conducted by the Anti‐Doping Laboratory Qatar (ADLQ) using its state‐of‐the‐art facilities and instrumentation. To meet the short reporting timeline of 24–72 h, additional staff was recruited, trained, and incorporated into the routine activities during the event period. Furthermore, six scientific experts from other World Anti‐Doping Agency (WADA)‐accredited laboratories were also invited to ensure adequate staffing throughout the event.

In total, 57 scientific and non‐scientific staff of ADLQ participated in the FIFA testing. A total of 1612 samples (623 urine, 622 serum, and 367 whole blood samples) were collected and analyzed. The number of serum samples that were tested for hGH using the isoform method had been the highest compared to any other sport event in the past. The results of all the analyzed urine and blood samples were negative, with two cases each being reported to contain the monitoring substance codeine and the confounding factor carboxy finasteride.

## Graphical abstract

The FIFA 2022 World Cup was fully accomplished by the Anti‐Doping Lab Qatar. The article describes the doping analysis work, achievements, and lab structure in details.

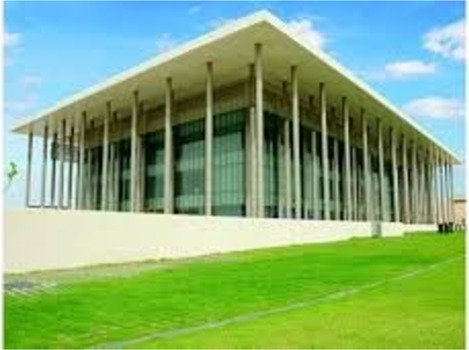

## 1 Introduction

The FIFA World Cup 2022, the world championship for national football teams, took place in Qatar from November 20th to December 18th, 2022, after the country was awarded the hosting rights in 2010. It was the first World Cup to be held in the Arab world, and the second to be held in Asia, following the 2002 tournament in South Korea and Japan. This tournament included 32 participating teams.

The athletes were subjected to both in‐competition and out‐of‐competition testing. In‐competition testing refers to urine or blood samples collected from 11:59 p.m. on the day before a competition until the competition ends for the athlete. Out‐of‐competition testing can occur at any other time during the tournament. This rigorous testing regime ensures that athletes are held to the highest standards before, during, and after the competition.

Doping control analysis is critical to maintaining fairness and integrity in sports competitions. The Anti‐Doping Laboratory Qatar (ADLQ), fully accredited by World Anti‐Doping Agency (WADA) since 2015, conducted the analyzes for all collected doping control samples, except for dried blood spots samples (DBS), that were transported for analysis to the Swiss Laboratory for Doping Analyzes (Lausanne, Switzerland). To prepare for the event, ADLQ implemented a rigorous training program for its staff, to ensure that they were adequately qualified to conduct all the required analyzes under the exceptional conditions of a big sport event with strict reporting timelines. In addition, a comprehensive risk assessment was performed to identify potential challenges and mitigate risks, in compliance with ISO 17025 and WADA's International Standard for Laboratories [1]. These measures were designed to streamline workflows, improve the accuracy and reliability of testing, and ensure that the laboratory operations will run smoothly throughout the event period. Samples were received from November 13th to December 18th, 2022.

The contract for the doping analysis for the event was signed between the Anti‐Doping lab Qatar and FIFA's Anti‐Doping Department. All analyzes were carried out with a state‐of‐the‐art technology and cutting‐edge analytical procedures to accurately identify the presence of banned substances [2]. These analytical procedures were mainly based on mass spectrometric (low or high resolution) detection combined with gas or liquid chromatography, as well as other analytical techniques based on immunoassays and gel electrophoresis.

## 2 Experimental

a Laboratory Accreditation

ADLQ is the only WADA‐accredited laboratory in the Gulf Cooperation Council (GCC) and one of six laboratories in Asia. ADLQ is accredited by NATA, Australia, for the ISO/IEC 17025 standard. Since FIFA World Cup testing is not classified as a Major Games event by WADA, additional assessment by WADA was not required.

b Facility and Security

ADLQ is an independent, dedicated facility located in Aspire Zone in Doha, Qatar. The building infrastructure is maintained in accordance with government regulations, and the laboratory has 24/7 independent security. Further, the laboratory was under CCTV surveillance and the permission to enter to each floor was controlled by card identification and corresponding authorization. Dedicated security personnel, including Fire, and Health, and Safety officers, have been on site to ensure the safety of the premises. The laboratory has its own certified staff clinic to manage any medical emergencies, and all staff received training in first aid before the FIFA World Cup.

c Chemicals and Materials

The certified reference materials were purchased from the National Measurement Institute (Australia), Toronto Research Chemicals (Canada), Laboratory of the Government Chemist (LGC), Sigma (United States) and Analytical One (Qatar). All other reagents, chemicals and gasses were purchased locally on an annual basis, to ensure the continuity of the lab operations. Immunopurification utensils for the analysis of erythropoiesis‐stimulating agents (ESAs) and human growth hormone (hGH) test kits sourced in ample amount, according to FIFA's test distribution plan. By means of an in‐house Reference Material Database software, ADLQ maintains reference materials in the laboratory.

d Laboratory Organization and Staff

The total number of staff from the routine lab (44) and research unit (7) contributed to the event, operated in three shifts, with minimal staff in the night shift to ensure uninterrupted running of the sequences on the LC and GC instruments. In addition, six experts from other WADA‐accredited laboratories were invited to ensure availability of sufficient expertise throughout the event.

e Logistics and Organization of Samples

Urine, blood, and DBS samples were delivered by the FIFA Doping Control Officers to the laboratory facility for registration into the Laboratory Information Management System (LIMS). The testing for urine and blood samples was carried out in ADLQ. At ADLQ, a designated laboratory team was responsible for the sample registration, aliquoting, and storage after measuring specific gravity (SG), and pH. The in‐house validated LIMS, which was customized to meet the laboratory's needs, was used to manage the sample chain of custody and reporting. At the time of sample receipt, all relevant details related to the samples, including additional analysis and consent for research, pH and SG for initial testing procedure (ITP) and confirmation procedure (CP) were manually entered into the Lab LIMS. The CSV file, which was generated from Lab LIMS, contained all the information required for reporting into the Anti‐Doping Administration and Management System (ADAMS), while the steroid profile concentration values and the information about the presence of confounding factors were manually entered into the CSV file before uploading it into ADAMS.

The lab ensured to perform requisite checks to verify that the information added in LIMS was accurate according to the documents received with the samples. The samples were double‐checked for the external codes against the accompanying Doping Control Forms (DCFs). During the entire process of sample movement—from aliquoting to storage‐ the process was verified by two scientists according to laboratory procedures.

After completing the registration and aliquoting, urine “A” samples were immediately prepared, while “B” samples were stored at −30°C. In the case of blood samples, both “A” and “B” samples were immediately centrifuged to obtain the serum fraction, with “A” being analyzed and “B” kept for storage at −80°C. The blood samples for the Athlete Biological Passport (ABP) were analyzed within 24 h of reception in the laboratory and were not subjected to storage at −80°C.

The laboratory operated with a well‐established backup system connected to all the lab equipment and internal share folders. All data was stored at three locations, i.e., internal server, hard disk backup, and cloud backup.

f Analytical Methods

For urine samples, the standard menu consisted of the following ITP:

○ Liquid Chromatography (LC)‐High Resolution Mass Spectrometry (LC‐HRMS)

Two screening LC‐HRMS procedures were routinely applied: liquid–liquid extraction (LLE) after hydrolysis with β‐glucuronidase for the detection of large number of analytes and a ‘Dilute‐and‐Shoot’ (DS) procedure for the detection of a limited number of analytes that were not adequately extracted or detected employing LLE. These were two separate lines of analysis (ITP) injected separately. Screening of more than four hundred substances was achieved by LC‐HRMS after enzymatic hydrolysis, followed by LLE. In brief, to a 5 mL aliquot of urine, 1 mL of phosphate buffer, (pH 7) was added, followed by 100 μL of β‐glucuronidase from *E. coli* and 50 μL of a mixture of internal standards (ISTDs). The samples were incubated for 1.5 h at 50°C and after the completion of hydrolysis, the pH was adjusted to 9–10 by the addition of solid carbonate buffer. The LLE was performed with 5 mL of ethyl acetate, utilizing anhydrous sodium sulfate as a salting‐out agent. After centrifugation, the organic layer was separated from the aqueous phase by freezing the samples at −80°C. The organic phase was then acidified with 200 μL of 3 M acetic acid in ethyl acetate and evaporated under a nitrogen stream at 50°C. The residue was reconstituted with 200 μL of reconstitution solvent and transferred to a vial in which 20 μL of unextracted urine were added [3].

○ Gas Chromatography (GC)—tandem mass spectrometry

The GC–MS/MS screening covered most of the exogenous and endogenous steroids, as well as compounds from various categories of prohibited substances. A 2.5 mL aliquot of urine was hydrolyzed using 50 μL of β‐glucuronidase enzyme from *E. coli* , followed by LLE. The solid‐phase extraction (SPE) was applied for samples with elevated pH values (> 8.0) to avoid hydrolysis inhibition since the SPE includes adjustment of pH at 7.0 for efficient hydrolysis by maintaining activity of the enzyme (β‐glucuronidase**)**. In the case of samples with a SG higher or equal to 1.024, the sample volume was reduced from 2.5 to 1 mL to avoid the saturation of the mass spectrometer detector for the analysis by GCQQQ especially for androsterone and etiocholanolone [4].

○ Immunoassay

All the samples were analyzed for human chorionic gonadotropin (hCG), and luteinizing hormone (LH) by Cobas e411 (Roche) and IMMULITE 1000 (Siemens), consecutively.

○ Additional analysis

The additional analysis for growth hormone releasing peptide (GHRP), and gonadotropin‐releasing hormone (GnRH) for urine samples was applied as per request received. For the analysis, 1 mL of urine was centrifuged and loaded in to preconditioned 1 cc 30 mg WCX cartridge, and washed with 1 mL of deionized water, eluted with 1 mL of elution solvent, then the eluate was evaporated in the vacuum centrifuge at temperature of 45^°^C for 1–2 h. After evaporation 100 μL of H_2_O was added to reduce the acidity of the sample and transferred to the vials and injected into the LC‐HRMS.

ESAs analysis was conducted using immunopurification on a 15 mL aliquot of urine samples, followed by gel electrophoresis in sarcosyl‐polyacrylamide gel electrophoresis (SAR‐PAGE) gel, single blotting, and revelation based on a chemiluminescence signal. As per the request received, the blood samples analysis for ABP, hGH isoform test and ESAs was conducted. The analytical equipment's used for the FIFA 2022 games are detailed Table 1.

**TABLE 1:** List of instruments available in the lab and the substances aimed to detect for the FIFA 2022 games.

Technique	Instrumental details	Number	Application	Substances	Matrix
GC–MS/MS	7000/7010 GC/MS Triple quad (Agilent technologies)	6	ITP/CP	Exogenous steroids Endogenous steroids	Urine
GC‐MSD	5977B GCMSD (Agilent technologies) 7890A GC system (Agilent technologies)	2	Peak identification for IRMS	Endogenous steroids	Urine
GC‐C‐IRMS	Isoprime	3	Steroid profile target compounds	Endogenous steroids	Urine
HPLC	LC 1290 Infinity (Agilent technologies)	1	Steroid profile target compounds	Endogenous steroids	Urine
LC‐HRMS	LC Ultimate 3000 Q Exactive (Thermo scientific)	5	ITP/CP	Small molecules Small peptides	Urine
LC‐QQQ/MS	LC 1290 Infinity (Agilent technologies) 6490 Triple quad LCMS (Agilent technologies)	2	Quantitative confirmation	Threshold substances	Urine
SAR PAGE	LAS‐4000 (ImageQuant)	1	ITP/CP	Erythropoiesis‐stimulating agents	Urine
					Plasma
					Serum
Immunoassay	IMMULITE 1000 (Siemens)	1	ITP	Human chorionic gonadotropin/luteinizing hormone	Urine
	Cobas e411 (Roche)	1	CP	Human chorionic gonadotropin	Urine
	AutoLumat LB953 (Berthold)	1	ITP/CP	Human growth hormone isoforms	Serum
Flow cytometry	Sysmex XN‐2000	1	ITP	Blood parameters	Whole Blood
Refractometry	ATAGO 3464	4	ITP/CP	Specific gravity	Urine
Potentiometry	Thermo orion star A111	2	ITP/CP	pH	Urine

## 3 Results and Discussion

**FIGURE 1:**
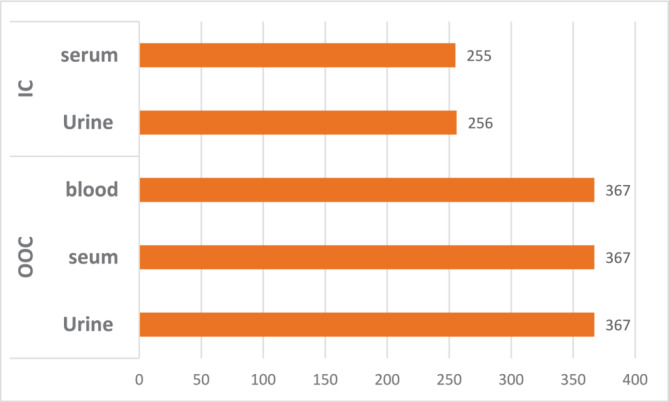
Total number of samples analyzed during IN and OUT of competition.

A total of 1612 urine and blood samples were analyzed for the FIFA 2022. Of these, 623 were urine samples and 622 were serum samples, all analyzed within 48–72 h. The blood samples for ABP (*n* = 367), collected during out of competition only, were analyzed within 24 h. A total of eight samples were analyzed by isotope ratio mass spectrometry (IRMS) during and after the event. In addition to the standard menu, additional tests were requested for both in‐competition and out‐of‐competition for blood and urine samples, including ABP, ESAs, GHRF, and hGH isoform. (Figure 1). All the urine samples with invisible ESA bands were subjected to blood ESAs analysis based on the laboratory's recommendation and in compliance with WADA TD2022EPO [5].

**FIGURE 2:**
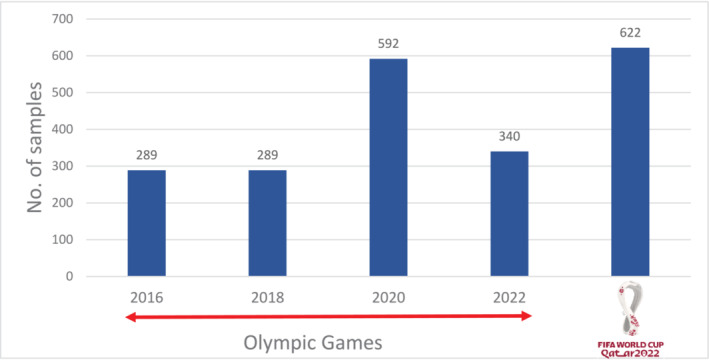
Comparison of hGH Isoform Tests performed in various Olympic games with FIFA 2022.

The Laboratory tested 622 samples for hGH Isoform test during the FIFA World Cup, which was the highest number of tests conducted by Anti‐Doping laboratory compared to the tests done during major games (summer and winter Olympics) [ 6, 7], as represented in Figure 2.

Quality assurance (QA) plays a pivotal role in maintaining the integrity and credibility of dope testing procedures in laboratories, which was followed at all levels, from sample reception to reporting. To ensure the integrity of drug testing, the laboratory implemented stringent quality control measures. The Quality Control (QC) samples were evaluated using QC charts throughout the period. The internal quality assurance samples (IQAS) were provided during the period (both threshold and non‐threshold substances) and the reported results were found to be accurate.

During the FIFA event, the Quality Team also verified all entries of doping control forms into the laboratory LIMS. Laboratory testing procedures included various negative and positive quality controls to ensure that the analysis meets the requirement outlined in the Standard Operating Procedure. Stringent criteria were observed throughout the evaluation of data. Each batch was reviewed by the two certifying scientists. Reporting on ADAMS is verified for each sample to ensure correctness of reported results.

The FIFA World Cup does not fall under the category of WADA Major events, so the laboratory did not receive any double‐blind samples with the FIFA samples. However, the laboratory correctly identified and reported two routine WADA Double‐blind samples which were received during the FIFA testing period.

The laboratory has a well‐established procedure to handle the findings related to therapeutic use exemption (TUE). However, throughout the event, no suspicious cases were observed that required a TUE inquiry. The analysis of all urine samples using LC and GC screening resulted in negative findings. However, two cases for the monitoring substance codeine were reported with high concentration, estimated in micrograms as per applicable WADA documents. The use of codeine intake was mentioned in the DCF of both the athletes. The presence of the confounding factor carboxy finasteride at an estimated concentration of higher than 5 ng/mL was found and the use of finasteride as medication was declared in the DCF in one of the two samples.

A total of 277 samples were analyzed for ESAs in urine and blood, which was higher in comparison to other events handled by ADLQ. The invisible band for ESAs was reported for seven samples which were later analyzed for ESAs in blood and reported negative.

- All samples analyzed for hGH were reported as negative. One case of hGH isoform exhibited a discrepancy in the ratio between REC/PIT on Kits 1 and 2; this case was referred for second opinion and was reported as negative. The projected number of samples for hGH isoforms, as outlined by FIFA in the TDP, created an opportunity for event planning, but also presented a significant challenge for the laboratory. In view of the availability of only one piece of equipment (Luminometer) for the analysis of hGH isoform test, a comprehensive risk assessment was performed. The laboratory implemented a series of actions to mitigate risks of the situation which were as follows:
- Contacted manufacturer, Berthold, to purchase another piece of equipment; however, the model was discontinued, and new model had not yet come to the market.
- Contacted laboratories in Tokyo, China, Brazil, and Korea, which handled major games (Summer and Winter Olympics), to secure a backup instrument, but all the labs had only one piece of equipment.
- The situation was informed to WADA and FIFA
- An application expert from Berthold was invited to the laboratory to ensure the proper functioning of the equipment and to perform preventive maintenance.
- Provision for subcontracting was made with the Swiss Laboratory for Doping Analyses, Lausanne in case of any adversity.
- Experts from London, Belgium, and China labs were invited to support hGH testing.

All the results were submitted through the ADAMS, except for a single ABP case with low MCH values. A paper report was issued, and ADAMS team was contacted to rectify the issue. The matter was taken up by the WADA APMU Manager. The sample report was later submitted through ADAMS once the issue was resolved. The case was described as a clinical condition which needs follow‐up from the testing authority.

A total of eight samples were analyzed by IRMS based on the notification through APMUs to investigate the exogenous origin of endogenous steroids as per WADA TDIRMS2022 [8]. All the samples were reported as negative.

## 4 Conclusion

The doping analysis for the FIFA World Cup 2022 was successfully accomplished through a meticulous preparation, adequate resources (workforce and instrumentation), staff training, quality control, analytical methods, facility, management of ADLQ, and support of FIFA organizing committee.

## Conflicts of Interest

The authors declare no conflicts of interest.

## Data Availability

Data sharing is not applicable to this article as no new data were created or analyzed in this study.
